# Urban Agriculture Enterprises and Food Security Outcomes in Secondary African Cities: Evidence From Mbarara City, Uganda

**DOI:** 10.1155/tswj/6930486

**Published:** 2026-06-11

**Authors:** Nabalegwa Muhamud Wambede, Turyahabwe Remigio, Arinaitwe Justine, Turyabanawe Loy, Mulabbi Andrew

**Affiliations:** ^1^ Department of Geography, Kyambogo University, Kampala, Uganda, kyu.ac.ug; ^2^ Department of Geography, Busitema University, Tororo, Uganda, busitema.ac.ug; ^3^ Department of Education, Uganda Christian University, Mukono, Uganda, ucu.ac.ug

**Keywords:** enterprise diversification, HFIAS, household food security, Uganda, urban farming, urban household

## Abstract

Urban agriculture is increasingly promoted as a strategy for enhancing household food security in African cities, yet empirical evidence from rapidly growing secondary cities remains limited, particularly regarding how different urban agricultural enterprises contribute to food access. This study examines the role of urban agriculture in shaping household food security in Mbarara City, Uganda, by comparing outcomes across arable, poultry, livestock and mixed farming enterprises. Using a cross‐sectional survey of 310 urban farming households, food security was assessed using the Household Food Insecurity Access Scale (HFIAS), and differences across enterprise types were analysed using descriptive statistics and chi‐square tests. The results indicate variation in food security outcomes across enterprise types. Households engaged in arable farming were more likely to be food‐secure than those relying primarily on poultry or livestock enterprises, whereas mixed‐enterprise households were associated with more stable food security outcomes, suggesting that diversification may buffer households against production‐ and market‐related risks. These differences reflect variations in land requirements, input costs and compatibility with dense urban environments. Overall, the study demonstrates that urban agriculture is associated with differing food security outcomes across enterprise types. By focusing on a secondary city context, the findings provide empirical evidence for debates on urban food systems and highlight the importance of enterprise selection and diversification in urban food planning amid rapid urbanisation and land constraints.

## 1. Introduction

Urban agriculture (urban and periurban agriculture [UPA]) includes all practises on land and in other urban spaces aimed at producing food and other agricultural outputs, as well as related processes such as transformation, recycling, distribution and marketing [1]. In doing so, it recycles the human and material resources, goods and services that are present in and around that urban area and, in turn, supplies human and material resources, goods and services primarily to that urban area globally [2, 3]. These activities encompass the cultivation, production, processing and distribution of a diverse range of food and nonfood products.

In developing and developed countries, UPA is driven by benefits such as promoting food security and alleviating poverty by providing jobs to the urban poor, including women [4]. This, in turn, enhances economic inclusion [5, 6]. It has been observed that particular nations have begun to recognise the potential advantages of UPA, including food security, environmental management and economic development [7]. Consequently, they have initiated the process of formulating policies that can guide this significant activity. The UPA has been demonstrated to facilitate a range of opportunities for urban residents to diversify their employment, income and dietary options, as well as to recycle and reuse urban waste [8, 9]. These actions contribute to sustainable urban development. UPA supports multiple Sustainable Development Goals (SDGs)—including Goals 1 and 2 by supporting livelihoods and increasing food access, SDG 3 through improved nutrition and well‐being, Goal 11 by strengthening urban resilience and land use, Goal 12 through localised and circular food systems and Goals 13 and 15 by supporting climate mitigation, adaptation and biodiversity—although achieving social, economic and environmental progress depends on addressing context‐specific challenges. Achieving social, economic and environmental progress aligned with the SDGs depends on addressing specific challenges [10]. Although these advantages are well known, less attention has been paid to how various types of urban agriculture operate within the institutional, financial and geographical constraints characteristic of rapidly expanding cities.

When strategically planned in urban environments, urban agriculture can be a highly effective approach to achieving sustainable food security for city dwellers, ensuring that each household has a sufficient supply of fresh food [11]. As such, urban structure plans increasingly need to incorporate provisions for urban agriculture as part of broader food system strategies to enhance food security. In this context, food security refers to a state in which all people have physical, social and economic access to sufficient, safe and nutritious food that meets their dietary needs and preferences for an active and healthy life [12]. Despite these benefits, rapid urbanisation in many African countries, averaging about 4% annually, has outpaced the development of infrastructure and food supply systems [13]. According to the United Nations [14], by 2035, over half of the African population will reside in urban areas, further intensifying the need for reliable and sustainable food systems. Although much research has focused on major capitals such as Nairobi, Accra and Lagos, smaller and secondary cities, where urban growth is often fastest, remain underrepresented in the literature [15–21]. This imbalance is significant because secondary cities are becoming increasingly important drivers of urban change in Africa. However, they often face their own problems, such as insufficient land, weaker policies and institutional frameworks and inadequate technical and financial support services for urban farmers [15, 16, 19].

It is imperative to enhance food provision for urban agriculture by safeguarding agricultural land in rural, periurban and urban regions. Furthermore, it is essential to identify novel locations for food production to establish sustainable food systems [22, 23]. The need for local food production to ensure a sustainable urban area is paramount. The local production of foodstuffs in urban areas has been demonstrated to reduce a nation′s reliance on the global food system, thereby reducing a region′s vulnerability to food supply [24]. Recent years have seen a marked increase in consumer demand for domestically produced food, driven by concerns over food safety, environmental sustainability and supply chain resilience. However, the type of agricultural enterprise chosen and its compatibility with urban land use, infrastructure and governance settings significantly affect how much these advantages materialise in practise [16].

Studies from other African countries have shown that periurban agriculture is a key element in sustaining the country′s urban food systems [15, 18, 19]. This is particularly evident in its support for the livelihoods of individuals residing in urban areas, with a focus on the economically disadvantaged. Urbanisation in East Africa is associated with an increase in the urban population, who in turn demand greater quantities of agricultural products, including poultry, dairy and fresh vegetables [25]. The majority of these products are produced in urban and periurban areas.

Even as studies of urban agriculture increase, much of this work treats it as a relatively uniform activity and provides little understanding of how various agricultural businesses contribute to household food security in urban settings. In practise, land needs, input prices, production cycles and susceptibility to market and disease risks vary significantly among urban agricultural businesses. Their food security outcomes will likely be influenced by these disparities, especially in secondary cities where formal planning frameworks are still developing and space is scarce. Therefore, an enterprise‐level perspective is crucial for guiding the development of urban food systems and for fostering sustainable, viable urban agricultural practises in such settings.

The unregulated nature of urban growth in Uganda is a salient issue that demands urgent attention. This has led to discordance in land use, with agriculture and urban development occurring in parallel. Within designated urban planning areas, the evolution of urban farming centres on the development of green spaces. Land designated for agricultural use is constrained [5, 26, 27]. In the rural periphery of urban areas, available agricultural land is competing with housing and industrial activities. The limited returns from agricultural output have led to the emergence of more lucrative urban activities, thereby driving the urbanisation of rural areas. In Kampala, the capital city of Uganda, there has been continuous encroachment on agricultural land for urban expansion, thereby reducing the available space for agricultural activities [27]. Individuals often show reluctance to allocate substantial resources to urban agriculture due to ambiguity regarding ownership and tenure rights [28]. Nonetheless, in informal settlements in Kampala City, small green spaces dedicated to urban agriculture serve as public areas for community skill training, livelihood activities and nonformal education. Urban authorities support these spaces [5, 29]. These land‐use pressures and tenure uncertainties create uneven conditions for different forms of urban agriculture, favouring enterprises that are less land‐intensive, more flexible and compatible with fragmented urban spaces.

Much of the early literature on UPA focuses on large, established cities, where policy frameworks and infrastructure have long shaped farming practises [30]. Although studies from cities in the Global North often emphasise institutionalised urban agriculture supported by secure tenure and formal planning frameworks, research from African cities highlights informal, space‐constrained practises shaped by rapid urbanisation and weak regulatory environments [31, 32]. Nevertheless, the fastest urban growth is now in smaller and medium‐sized cities, particularly on the periurban fringes, where land‐use pressures, rapid population growth and resource constraints shape unique food production systems [33–35]. Despite their potential, these cities remain underrepresented in research, with recent studies confirming that local food sourcing varies by city size, markets and location [21, 36] and calling for stronger integration of UPA into early‐stage municipal planning [17]. Although these studies highlight the importance of urban agriculture in smaller and medium‐sized cities, they provide limited empirical insight into how different types of urban agricultural enterprises contribute to household food security and which enterprise forms are most compatible with the socio‐spatial conditions of secondary cities.

Understanding food security dynamics in secondary cities is critical because these urban centres now absorb a large share of Africa′s urban population growth, yet receive comparatively limited policy attention and infrastructure investment. Mbarara City, Uganda′s second largest urban centre, illustrates this gap. With fertile soils, favourable altitude and the River Rwizi for irrigation, urban farming, mainly subsistence arable, poultry and livestock keeping is common, yet its contribution to household food security is undocumented. Although urban agriculture in capitals like Nairobi, Accra and Lagos has been studied extensively [23, 37–39], limited empirical work exists on secondary cities, and existing research often relies on descriptive rather than standardised measures [40, 41]. This limits policy guidance on which enterprises most effectively promote food security and reduce poverty [42]. These characteristics make Mbarara City a suitable case for examining how different urban agricultural enterprises shape household food security outcomes in rapidly growing secondary cities in Africa. By adopting an enterprise‐level perspective and standardised food security measurement in a secondary city context, this study responds directly to recent calls for more differentiated and policy‐relevant analyses of urban agriculture. This study is among the first to empirically compare food security outcomes across urban agricultural enterprise types in a rapidly growing secondary city using a standardised food access metric.

Based on this background, this study addresses the following research questions: (1) How do household food security outcomes vary across different urban agricultural enterprises in Mbarara City? (2) Which forms of urban agriculture are most compatible with dense urban environments in terms of food security outcomes? (3) What are the implications of enterprise‐specific food security outcomes on urban food system planning in secondary cities?

## 2. Materials and Methods

### 2.1. Description of the Study Area

**Figure 1:**
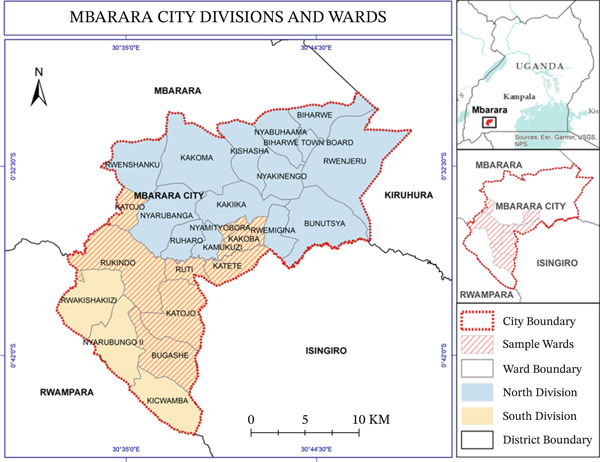
Map of Mbarara City showing the location of the South Division and its wards.

The study was conducted in the South Division of Mbarara City, which is located in Southwestern Uganda. Mbarara City is advantageously located as a major economic hub along the highway to Rwanda and the Democratic Republic of the Congo. It is the most populous city in southwestern Uganda. Mbarara City, situated in the south‐western part of Uganda, is located 266 km from Kampala on the Kampala–Kabale road. Indeed, it is found at longitude 30037I East and latitude 0036I South. The city functions as the primary economic hub and is home to the Mbarara District′s political and administrative centres. The total land area within the municipal boundaries is approximately 449.09 km^2^ (44,909 ha), further divided into two districts under consideration in this study: Mbarara City South and Mbarara City North, with areas of 191.49 and 257.6 km^2^, respectively. The urban district of Mbarara City comprises 187 villages and 23 wards (Figure 1).

The city′s topography is characterised by hilly terrain interspersed with brief, narrow and typically shallow valleys. The city is located on a high plateau that forms part of the East African Rift Valley, at an average elevation of 1432 m above sea level. Mbarara City experiences a bimodal rainfall pattern with two distinct rainy seasons and two dry seasons. The mean annual temperature is 25^°^C, and the mean annual rainfall is 1125 mm [43]. The city is characterised by a range of soil types, including clay loams, sand loams and marram. The predominant vegetation is open savannah grassland interspersed with woodland.

In recent decades, Mbarara City has seen substantial changes in both its population and its physical layout. The city is the central urban hub in southwestern Uganda, with an estimated population of about 280,000, according to the Uganda Bureau of Statistics (UBOS) [44]. Its significance as a regional administrative, economic and educational centre, rural–urban migration and the city′s elevation in 2020 all contribute to this population increase.

Because of its combined urban and periurban nature, the city is home to a variety of economic activities. Wholesale and retail commerce, logistics and transportation, real estate and financial services are the main drivers of the formal economy. UPA, small‐scale trading and artisanal services are among the subsistence‐level and informal jobs that a significant portion of the population pursues [45, 46]. Particularly in informal settlements and outlying locations where land access is more practical, urban agriculture is essential to improving food security, income diversification and employment for both newcomers and long‐term inhabitants [47]. Mbarara City′s rise as a regional agribusiness hub has been accelerated by its advantageous location along key transit routes, which facilitate the transport of horticultural, dairy and banana products to both local and international markets. Mbarara is a relevant case study for researching the role of urban agriculture in rapidly growing secondary cities, given its dynamic urban–rural interactions.

### 2.2. Classification of Urban Agricultural Enterprises

The study classified urban agricultural enterprises into four categories: arable (food crop) farming, poultry farming, livestock farming and mixed enterprises. This classification is consistent with established UPA literature, which commonly distinguishes between crop‐based systems, animal‐based systems and diversified production strategies based on differences in land requirements, capital intensity, labour demand and risk exposure [37, 42, 48]. Crop‐based (arable) urban agriculture is typically characterised by relatively low capital requirements, short production cycles and high compatibility with small and fragmented urban spaces, making it a common livelihood strategy among low‐income urban households [49, 50]. Poultry and livestock enterprises, in contrast, generally require higher input costs, specialised management and greater space and are more susceptible to disease outbreaks and market price fluctuations, particularly in dense urban environments [51, 52].

Mixed enterprises were treated as a distinct category because diversification across crops and animals is widely recognised as a risk‐buffering strategy that enhances food availability, dietary diversity and income stability in urban and periurban settings [9, 15]. Analysing mixed enterprises separately enables an assessment of whether diversification yields different food security outcomes than specialised enterprise types. This enterprise‐level classification provides an analytically meaningful framework for examining how different forms of urban agriculture interact with urban space constraints and contribute to household food security outcomes in secondary cities.

### 2.3. Sampling and Data Collection Methods

The study adopted a cross‐sectional design and was conducted in the South Division of Mbarara City, comprising seven wards, including Kakoba, Ruti, Nyamityobora, Katete, Bugashi, Rukindo and Katojo, with a total of 1374 households across 64 cells [44]. The division was purposively selected for its concentration and diversity of urban agricultural enterprises. A sample of 309 households was determined using Slovin′s formula and a multistage sampling technique. First, a stratified sampling design was employed, with each ward treated as a stratum. Secondly, cells were selected proportionally to the number of households in each ward, yielding 31 cells. Thirdly, households were selected from these cells using simple random sampling. To ensure adequate representation across different types of urban agriculture enterprises, households were further grouped into four subgroups: crop‐based, livestock‐based, poultry‐based and mixed enterprises. The number of households selected from each subgroup was determined proportionally to their prevalence in the study area. Therefore, of the 309 sampled households, 133 are engaged in crop production, 83 in livestock production, 60 in poultry production and 33 in mixed enterprises. By doing so, we ensured that all major enterprise types were represented, enabling comparative analysis of food security outcomes across subgroups. Data collection took place between October 2023 and February 2024, with information on urban agricultural enterprises (crops, livestock, poultry and mixed enterprises) collected through structured questionnaires, focus group discussions, interviews and field checklists. The questionnaire was available in English and in a Runyankole‐Rukiga translation, and included both closed‐ and open‐ended items. The urban household farmers were asked about the effect of different urban agricultural enterprises, and their food access status was assessed using the Household Food Insecurity Access Scale (HFIAS) (Appendix 1). The indicators of food access included those standardised and outlined in the table below, as outlined by [53]. A pilot test was conducted to refine the tool prior to the primary survey. Although focus group discussions, interviews and field checklists informed contextual understanding and instrument refinement, the quantitative analysis presented in this paper is based on the structured household questionnaire.

### 2.4. HFIAS Score

The HFIAS was used to assess household food security because it is a well‐validated, context‐sensitive measure widely applied in low‐ and middle‐income urban settings where food insecurity is often episodic [53, 54]. The scale captures gradations in food access that are particularly relevant to urban livelihoods and enables comparisons across different types of urban agricultural enterprises. The HFIAS consists of nine occurrence questions reflecting the increasing severity of food access constraints experienced during the 4 weeks preceding the survey (Appendix 1). Each question is scored as ‘never’ (0), ‘rarely’ (1), ‘sometimes’ (2) or ‘often’ (3). The total HFIAS score is calculated as the sum of all nine items, yielding a possible range from 0 to 27, with higher scores indicating greater food insecurity (Equation 1).

(1) HFIAS score=Y1+Y2+Y3+Y4+Y5+Y6+Y7+Y8+Y9

where *Y*_1_, *Y*_2_, *Y*_3_, *Y*_4_, *Y*_5_, *Y*_6_, *Y*_7_, *Y*_8_ and *Y*_9_ are occurrences of events stated in questions shown in HFIAS score table (Appendix 1).

To classify household food security status, the total HFIAS score, which ranges from 0 to 27, was used, with higher scores indicating greater food insecurity [53]. Although the original HFIAS developed by Coates et al. [53] does not prescribe fixed numerical thresholds for categorising households, this study adopted score‐based groupings for analytical clarity and comparability with previous empirical applications in urban contexts [41, 54]. Accordingly, households were grouped into four categories: food‐secure (0–6), mildly food‐insecure (7–13), moderately food‐insecure (14–20) and severely food‐insecure (21–27). These intervals approximate increasing severity of food access constraints, ranging from anxiety and reduced dietary quality to decreased food intake and experiences of hunger, and facilitate consistent comparison of food security outcomes across urban agricultural enterprise types.

### 2.5. Data Analysis

Data were analysed using SPSS Version 16.0. We first used descriptive statistics (frequencies and percentages) to summarise household characteristics, types of urban agricultural enterprises and food security status. Secondly, the HFIAS scores were analysed both as continuous measures and as categorised food security levels derived from score‐based groupings for comparative analysis. Associations between food security status and urban agricultural enterprise types were examined using Pearson′s chi‐square tests, given the categorical nature of the variables. This approach enabled comparison of food security outcomes across enterprise types and wards. Statistical significance was assessed at the 5% level (*p* < 0.05). Chi‐square tests were selected because they are appropriate for examining associations between categorical food security outcomes and enterprise types; however, these tests do not imply causation.

In addition to aggregate HFIAS scores, the frequency‐based response categories of the HFIAS items (‘rarely’, ‘sometimes’ and ‘often’) were used to capture differences in the intensity of food insecurity experiences across households. These categories reflect the increasing severity of food access constraints and are central to the HFIAS conceptual framework [53]. Chi‐square tests were applied to examine whether the distribution of these frequency categories varied significantly across urban agricultural enterprise types and wards, thereby allowing assessment of both the prevalence and intensity of food insecurity in relation to enterprise choice.

### 2.6. Ethical Consideration

The study adhered to ethical principles for social research involving human participants. Verbal informed consent was obtained from all respondents prior to data collection, and participation was voluntary. Respondents were informed of the study′s purpose and assured of anonymity and confidentiality. At the time of data collection, formal ethical clearance was not required by the University′s Directorate of Graduate Training and Research, which had granted an institutional waiver due to the absence of a research ethics committee. No personal identifiers were collected.

## 3. Results

### 3.1. Characterisation of Urban Agricultural Enterprises Practised in the South Division of Mbarara City

**Figure 2:**
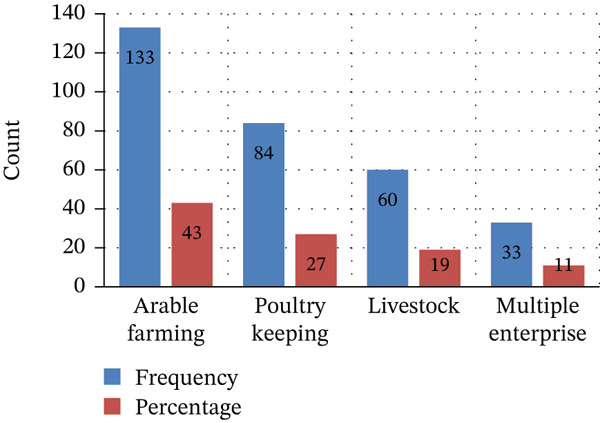
Distribution of urban agricultural enterprises in the South Division of Mbarara City.

Arable farming in Mbarara City was the most prevalent, practised by 43% of urban farmers (Figure 2). The dominant crop was bananas, cultivated mainly in backyards for both food and income. Other crops grown included vegetables such as cabbage, Sukuma wiki (spinach), tomatoes, onions, carrots and beans, often grown in sacks, polythene bags, ridges, rooftops and verandas. In some households, fruit trees, including jackfruit and avocado trees, were planted in backyards to supplement income and dietary needs. The dominant vegetable‐growing wards included Kakoba, Katete and Ruti. Farmers reported limited perceived impacts of climate variability on small domestic gardens, partly because they could irrigate their crops with water from the River Rwizi, boreholes, tap water and wells. Farm sizes ranged from a few square metres in the city core to several hectares on the outskirts. Most agricultural labour was provided by household members, especially women and children.

Livestock farming accounted for 19% of enterprises (Figure 2). Goats, cattle, pigs and rabbits were the most common species, raised in backyard enclosures or larger kraals on the periphery. Additionally, poultry farming dealing in both local and exotic breeds (broilers and layers) ranked second at 27% (Figure 2). Free‐range systems were frequent in peripheral areas, whereas confined systems dominated in the city centre due to space constraints and theft risks. The avian collection includes both indigenous and local species, such as ducks, doves, guinea fowls and most notably, chickens. These supplement the urban food systems in the form of eggs and meat. Multiple enterprises were also common, where some households combined arable farming, poultry and livestock rearing to increase the availability and diversity of food within limited urban space. Spatial limitations were commonly reported as constraining the number of enterprises households could operate.

### 3.2. Food Security Outcomes by Urban Agricultural Enterprises in Mbarara City

**Figure 3:**
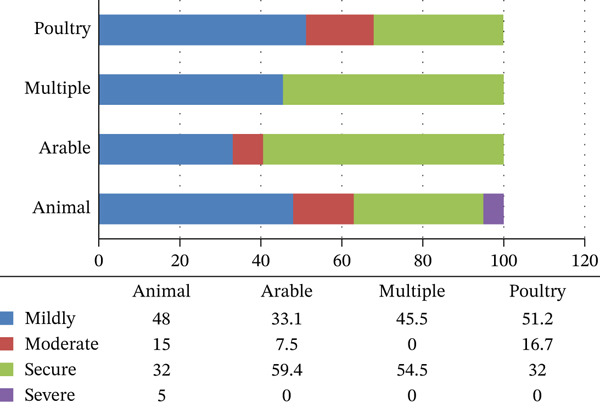
Food security status by urban agricultural enterprise across wards in South division, Mbarara City.

Of the 133 farmers engaged in arable farming, 59.4% were food secure. The majority of this percentage was from Katete and Nyamityobora (Figure 3). The study revealed that 33% of arable farmers experienced mild food insecurity, whereas no households reported severe food insecurity. Among poultry farming households, 51.2% experienced mild food insecurity, whereas 32% were food secure (Figure 3). Similarly, among the urban livestock farmers engaged, 32% reported being food secure, whereas 48% were mildly food insecure. Livestock‐based households showed a statistically significant association with higher levels of food insecurity at the 5% level.

In Mbarara City, farmers engaged in multiple urban farming enterprises had the most secure outcome, reporting food security and mild food insecurity. Despite their smaller numbers, households engaged in multiple enterprises were associated with the most favourable food security outcomes. Across the entire sample of urban farmers in Mbarara City, the study indicates that the majority (46%) are food secure, whereas 42% are mildly food insecure and 11% are moderately food insecure. Only 1% of households in Mbarara city are currently experiencing severe food insecurity, confirming relatively low levels of acute vulnerability. These figures are based on the aggregate totals of all the farmers involved in the study. In general, the majority of farmers who were food‐secure or mildly food‐insecure practised arable farming, whereas severe food insecurity was associated only with animal and livestock farming (Figure 3). These results show that food security outcomes vary significantly across urban agricultural enterprise types, suggesting that enterprise choice is linked to family food availability in Mbarara city.

### 3.3. Ward‐Level Differences in Household Food Security Status

Ward‐level variation in food security indicators was examined using binomial tests and Pearson′s chi‐square tests. Table 1 summarises associations between urban agricultural enterprises and individual food security indicators.

**Table 1:** Association between urban farming enterprises and food security indicators.

Food security indicator	Arable farming (*p*)	Poultry farming (*p*)	Livestock farming (*p*)	Multiple enterprises (*p*)	Significant associations
Any worry about enough food?	0.001 ✓	0.286 *x*	0.151 *x*	0.116 *x*	Arable only
Is any member unable to eat their preferred food?	< 0.001 ✓	< 0.001 ✓	0.001 ✓	0.025 ✓	All types
Any member ate a limited variety of food?	< 0.001 ✓	< 0.001 ✓	< 0.001 ✓	0.025 ✓	All types
Any member ate disliked food?	0.086 *x*	*p* < 0.001 ✓	0.141 *x*	0.462 *x*	Poultry only
Did any member eat a smaller amount of food?	< 0.001 ✓	0.003 ✓	0.195 *x*	< 0.001 ✓	Arable, poultry, multiple
Did any member have to eat fewer meals per day?	< 0.001 ✓	0.160 *x*	< 0.001 ✓	< 0.001 ✓	Arable, livestock, multiple
Ever no food to eat in the household?	< 0.001 ✓	< 0.001 ✓	< 0.001 ✓	< 0.001 ✓	All types
Has any member gone to bed hungry?	< 0.001 ✓	< 0.001 ✓	< 0.001 ✓	< 0.001 ✓	All types
Has any member gone a whole day and night without eating?	< 0.00 √	< 0.001 ✓	< 0.001 ✓	< 0.001 ✓	All types

*Note:* √: Statistically significant (*p* < 0.05); x: not significant.

For households engaged in arable farming, eight of the nine food security indicators showed statistically significant variation (*p* < 0.05), except for the consumption of disliked food (*p* = 0.086). Poultry farming households showed significant differences for eight indicators, with food sufficiency and reduced meal frequency not differing significantly from the reference value. Livestock‐based enterprises showed statistically significant variation in six indicators, whereas food sufficiency and disliked food did not vary significantly (*p* > 0.05). In multiple‐enterprise households, eight indicators were significant, whereas food sufficiency and consumption of disliked foods showed no significant differences (Table 1).

A second stage of analysis employed chi‐square tests to examine variation in categorical food security responses across wards. Categorical responses (rarely, sometimes and often) were used. These results are summarised in Table 2. For arable farming households, food security outcomes were largely consistent across wards, though significant differences emerged for preferred food access (*p* = 0.015) and consumption of disliked food (*p* = 0.040). In poultry households, no significant differences were observed across wards (*p* > 0.05). Among livestock households, food variety differed significantly across wards (*p* = 0.042), whereas other indicators remained consistent. Households engaged in multiple enterprises were associated with generally uniform food security outcomes across wards, with no statistically significant differences observed (*p* > 0.05).

**Table 2:** Chi‐square test analysis results of the occurrence of food security across all seven wards in the South Division of Mbarara City.

Second step: food security occurrence indicators	Food security occurrence in different wards						
	Rarely	Sometimes	Often	Total	Person′s chi‐square	Df	*p*‐value
Urban arable farming enterprise							
Did you worry about not having enough food?	46	5		51	5.839^b^	6	0.442
Household member not able to eat preferred food?	48	18		66	15.775^b^	6	0.015
Did the household eat a limited variety?	43	3		46	11.770^b^	6	0.067
Did a household member eat the disliked food?	13			13	13.194^b^	6	0.040
Did a household member eat a smaller amount?	4			4.0			

Urban poultry farming enterprise							
Did you worry about not having enough food?	46	30		76	9.305^d^	6	0.157
Household member not able to eat preferred food?	44	33		77	5.411^d^	6	0.492
Did the household eat a limited variety?	56	13		69	4.038^d^	6	0.672
Did a household member eat the disliked food?	56	5		61	3.782^c^	6	0.706
Did a household member eat a smaller amount?	14	1		15	4.286^c^	4	0.369

Urban animal/livestock farming enterprise							
Did you worry about not having enough food?	39	11	1	51	16.071^a^	12	0.188
Household member not able to eat preferred food?	57	15		72	8.877^a^	6	0.181
Did the household eat a limited variety?	53	9		62	13.053^a^	6	0.042
Did a household member eat the disliked food?	28	5		33	8.067^a^	6	0.233
Did a household member eat a smaller amount?	23			23	4.553^a^	4	0.336

Urban multiple farming enterprise							
Did you worry about not having enough food?	20	3		23	6.469^c^	4	0.167
Household member not able to eat preferred food?	22	3		25	7.244^c^	4	0.124
Did the household eat a limited variety?	20			20	2.214^c^	4	0.697
Did a household member eat the disliked food?	3			3			
Did a household member eat a smaller amount?	1			1			

*Note:* Superscripted letters a–d are SPSS‐generated footnote markers indicating chi‐square test diagnostic information associated with expected cell frequencies. Interpretation of results is based on the reported Pearson′s chi‐square statistics, degrees of freedom (df), and *p* values.

## 4. Discussion

The dominance of arable farming in Mbarara City and its association with comparatively higher food security outcomes are consistent with the suitability of crop‐based systems for space‐constrained urban environments. This pattern mirrors trends observed across many African towns, where urban crop cultivation plays a central role in household food provisioning and income generation. In Mbarara, bananas and a range of vegetables constitute the core of urban crop production, although the specific crop mix varies across cities in response to dietary preferences, cultural practises and agroecological conditions, as observed in Kisumu and Thika [55]. Vegetables are frequently produced in highly constrained spaces using sacks, rooftops and verandas, demonstrating adaptive responses to land scarcity, a pattern similarly reported in Windhoek [56, 57].

Fruit trees further contribute to dietary diversity and supplemental income, whereas wards such as Kakoba, Katete and Ruti illustrate the potential of microgardening to enhance urban nutrition. The perceived reliability of these systems is reinforced by access to irrigation from the River Rwizi and other local water sources, which may reduce sensitivity to short‐term climatic variability. However, the heavy reliance on household labour, particularly from women and children, raises concerns regarding the long‐term sustainability and equity of these practises. Taken together, these characteristics may help explain the observed association between crop‐based enterprises and better food security outcomes compared with livestock and poultry under land‐constrained urban conditions.

The prevalence of poultry farming as the second most prevalent farming enterprise in Mbarara City (27%) demonstrates its utility as an adaptable and accessible type of urban agriculture. The prevalence of exotic breeds such as broilers and layers in the city centre is attributable to limited land availability, as these breeds are more productive in confined systems and offer faster returns in the form of eggs and meat. Conversely, the use of local breeds in peripheral locations indicates both cultural preference and access to larger plots that can sustain free‐range systems. Comparable spatial trends have been reported in Pretoria, where small‐scale farmers in densely populated urban areas use confined poultry units, whereas those in peripheral locations keep free‐range flocks [58]. Although exotic breeds facilitate market‐oriented production, they also subject farmers to higher feed costs and greater disease vulnerability, as documented in studies conducted in Accra and Nairobi [51, 52]. Local breeds, however, are more resilient but offer lower productivity and are associated with lower food security outcomes. These findings suggest that although poultry farming contributes to urban food systems, its benefits for food security are constrained by costs, disease exposure and spatial limitations.

Livestock‐based urban farming enterprises in Mbarara were associated with comparatively weaker food security outcomes, mainly due to structural and economic constraints inherent to dense urban environments. Livestock production is characterised by high recurrent costs, particularly for feed and veterinary care, alongside limited space for housing animals, which constrains herd size and productivity. Similar challenges have been documented in cities such as Accra, Nairobi and Tamale, where escalating feed prices, disease outbreaks and restricted land access reduced the net food and income benefits of urban livestock keeping [42, 52]. Despite these limitations, livestock enterprises continue to play a supplementary role in urban food systems by providing access to animal‐source proteins such as meat and milk, which are critical for household nutrition. However, when practised as a sole enterprise under urban constraints, they were associated with lower overall household food security outcomes. These constraints help explain the relatively poorer food security outcomes associated with livestock‐dependent households in compact urban settings.

The comparatively better food security outcomes associated with multienterprise households may reflect the role of diversification as a risk‐buffering strategy in land‐ and market‐constrained urban environments. Combining arable crops with poultry or livestock can potentially enhance access to both staple and protein‐rich foods and may reduce exposure to production‐ or disease‐related shocks. These patterns align with findings from Addis Ababa, where diverse urban production spanning crops and animals enhanced food supply and access [15]. More broadly, multienterprise farming has been associated with improved resilience in low‐ and middle‐income cities through its contributions to both nutrition and household income [9]. However, the practise remains relatively limited in Mbarara because of land shortages and competing urban land uses. This contrasts with experiences in places such as Gent, Germany, where beneficial land tenure regimes and long‐established planning assistance have enabled varied firms to dominate and ensure the highest levels of family food security [59]. These contrasts highlight the importance of policy approaches in secondary African cities that support small‐scale diversification through secure land access, space‐efficient methods and integrated planning frameworks.

Ward‐level analysis further revealed that food security outcomes are linked not only to enterprise type but also to geographical location. Food quantity and dislike for certain foods varied significantly across wards, whereas concerns about food sufficiency were generally uniform. These differences may reflect variations in soil fertility, market access and input availability [60, 61]. In contrast, poultry farmers reported uniform outcomes across wards, likely because they face similar challenges with feed costs and market access, consistent with findings in Nigeria where smallholder producers exhibited uniform food outcomes despite geographical disparities [62–65]. Differences in the amount of food consumed among livestock‐keeping households may reflect variability in feed prices and space, as also reported in Nairobi [66]. Together, these patterns indicate that enterprise type interacts with local spatial and market conditions, reinforcing the need for enterprise‐level interventions to be complemented by location‐specific urban planning measures that address uneven access to land, inputs and markets in secondary cities.

This study demonstrates that food security outcomes are not uniform across urban agriculture households; rather, they vary by enterprise type and spatial context. Although poultry and livestock enterprises may benefit from focused investment in feed supply chains, disease management and space‐efficient production techniques to address their current constraints, arable farming is consistently associated with comparatively higher observed levels of food security. Poultry farming should be accorded targeted support. By increasing food diversity and decreasing susceptibility to shocks, promoting diverse farming operations may improve household resilience. In general, including urban agriculture in secondary city design has the potential to enhance household nutrition, reduce reliance on external food supplies and advance the SDGs of poverty alleviation, hunger alleviation and sustainable cities.

The present study is limited by its cross‐sectional design, which does not allow for establishing a causal relationship between household food security and the type of urban agricultural enterprise. It is hypothesised that the observed relationships may be attributable to other unmeasured characteristics, including but not limited to household income and market access. Moreover, the use of self‐reported food security data from the HFIAS may introduce biases, including recall bias and social desirability bias. The present study was conducted over a single agricultural season, thus disregarding the potential impact of seasonal variations in food availability, market pricing and output. Food security in Uganda is known to fluctuate significantly between the rainy and dry seasons. Consequently, this study provides only a snapshot of the situation, rather than year‐round conditions. Additionally, using the chi‐square analysis alone limits the scope of the findings to statistical associations and does not permit interpretations on the direction and magnitude of relationships. Further, using the HFIAS, which focuses on the food access dimension, misses out on capturing other equally important food security dimensions, such as dietary diversity and nutritional quality.

Notwithstanding the limitations aforementioned, the results provide important insights into the role of urban agriculture in rapidly growing secondary cities. The association between arable farming and enhanced food security is well documented; therefore, it can be hypothesised that public support for small‐scale crop production may be linked to improvements in family well‐being, even in urban settings where land is scarce. Moreover, the substandard performance of the livestock and poultry industries in ensuring food security highlights the importance of targeted interventions, including enhanced feed supply chains, disease control measures and market integration strategies. Secondary cities such as Mbarara continue to expand swiftly, yet receive fewer policy interventions than capital cities. As such, incorporating urban agriculture into early‐stage city planning and development may help these urban centres move toward more resilient and self‐sufficient food systems, reduce reliance on erratic external food sources and improve the standard of living for low‐income households. Such strategies are particularly relevant for secondary cities facing rapid population growth and constrained resources to eradicate hunger, reduce poverty and create sustainable cities, especially where these pressures are most pronounced.

## 5. Conclusions

This study shows that household food security outcomes vary across urban agriculture households in Mbarara City, with differences observed by enterprise type. Arable farming, particularly backyard crop production, was associated with better household food access, whereas poultry and livestock enterprises showed more limited food security benefits in dense urban settings. Engagement in multiple agricultural enterprises, although limited by land availability, was associated with comparatively better food security outcomes, suggesting the potential importance of diversification in mitigating production and market risks.

These findings highlight the need for urban food planning frameworks in secondary cities to move beyond viewing urban agriculture as an informal or marginal activity. Municipal policies could prioritise support for small‐scale arable farming by ensuring secure access to land, water and extension services, while addressing the structural constraints faced by poultry and livestock producers. Targeted interventions, such as improved veterinary outreach, affordable and reliable feed supply chains and space‐efficient production systems, may improve food security outcomes of animal‐based enterprises without exacerbating land‐use pressures. Complementary measures, including farmer training in pest and disease management, composting of organic waste, adoption of climate‐resilient crop varieties and investment in water‐harvesting technologies, may further support the sustainability of the urban food systems. Overall, the study highlights urban agriculture as a practical entry point for supporting food security and resilience in rapidly urbanising secondary cities. Integrating urban agriculture into early‐stage urban planning and food system strategies may help reduce reliance on external food supplies and support more inclusive, sustainable urban development pathways in Uganda and similar sub‐Saharan African contexts.

## Author Contributions

N.M.W.: conceptualisation, writing—original draft and supervision; T.R.: methodology, writing—original draft and formal analysis; A.J.: investigation, data curation and formal analysis; T.L.: supervision and writing—review and editing; M.A.: writing—review and editing, visualisation and formatting.

## Funding

No funding was received for this manuscript.

## Conflicts of Interest

The authors declare no conflict of interest.

## Supporting information


**Supporting Information** Additional supporting information can be found online in the Supporting Information section.

## Data Availability

The data generated and used in this study can be made available from the first author upon reasonable request.
