# Supplementary material for: Urban Agriculture Enterprises and Food Security Outcomes in Secondary African Cities: Evidence From Mbarara City, Uganda
Source: ScientificWorldJournal. 2026 Jun 11;2026:6930486. doi: 10.1155/tswj/6930486 (PMC13260864; doi:10.1155/tswj/6930486)
Supplement: Supplementary file 1 — Supporting Information Additional supporting information can be found online in the Supporting Information section. [file TSWJ-2026-6930486-s001.docx]

Appendix 1. *HFIAS* Occurrence questions from which two indicators were used to find out the food access status of households. For this study, the HFIAS score was adopted.

| **No.** | **Occurrence Questions** |
| --- | --- |
| 1 | In the past four weeks, did you worry that your household would not have enough food? |
| 2 | In the past four weeks, were you or any household member not able to eat the kinds of foods you preferred because of a lack of resources? |
| 3 | In the past four weeks, did you or any household member have to eat a limited variety of foods due to a lack of resources? |
| 4 | In the past four weeks, did you or any household member have to eat some foods that you really did not want to eat because of a lack of resources to obtain other types of food? |
| 5 | In the past four weeks, did you or any household member have to eat a smaller meal than you felt you needed because there was not enough food? |
| 6 | In the past four weeks, did you or any household member have to eat fewer meals in a day because there was not enough food? |
| 7 | In the past four weeks, was there ever no food to eat of any kind in your household because of a lack of resources to get food? |
| 8 | In the past four weeks, did you or any household member go to sleep at night hungry because there was not enough food? |
| 9 | In the past 4 weeks, did you or any household member go a whole day and night without eating anything because there was not enough food? |
